# Vulnerability and risk management of *Agave* species in the Tehuacán Valley, México

**DOI:** 10.1186/1746-4269-10-53

**Published:** 2014-07-03

**Authors:** América Delgado-Lemus, Ignacio Torres, José Blancas, Alejandro Casas

**Affiliations:** 1 Laboratorio de Ecología y Evolución de Recursos Vegetales, Centro de Investigaciones en Ecosistemas, (CIECO), Universidad Nacional Autónoma de México, Apartado Postal 27-3, Santa María Guido, C.P. 58090 Morelia, Michoacán, Mexico

**Keywords:** Agave, Ecological ethnobiology, Non-timber forest products, Risk management, Sustainability, Tehuacán Valley, Vulnerability

## Abstract

**Background:**

Our study analysed the vulnerability of the useful *Agave* species of the Tehuacán Valley, Mexico, considering ecological, cultural and economic aspects, and management types. We hypothesized that management intensity is proportional to the degree of risk of a species in order to decrease its vulnerability.

**Methods:**

Distribution of *Agave* species was monitored in 36 types of plant associations. Ethnobotanical studies were conducted in 13 villages and six markets. The vulnerability of each species was calculated by assigning risk values to the variables analysed. The vulnerability and management intensity indexes were estimated through the scores of the first principal component of PCA. Variation of management data explained by ecological, cultural and economic information were analysed through canonical correspondence analyses (CCA). A linear regression analysis identified the relation between vulnerability and management intensity.

**Results:**

We recorded presence of agave species in 20 of 36 vegetation types. Out of 34 *Agave* species, 28 were recorded to have one to 16 use types; 16 species are used as food, 13 for live fences, 13 for producing ‘pulque’, 11 for fibre and ornamental, 9 for construction. Seven species are used for preparing mescal, activity representing the highest risk. Seven *Agave* species are exclusively extracted from the wild and the others receive some management type. Incipient cultivation was identified in *A. potatorum* whose seedlings are grown in nurseries. Intensive cultivation through vegetative propagation occurs with domesticated species of wide distribution in Mexico. The highest management intensity values were recorded in widely distributed, cultivated and domesticated species, but the regionally native species more intensively managed were those with higher demand and economic value, protected by collective regulations because of their scarcity. The regression analysis indicated significant relation (R^2^=0.677, *P*<0.001) between vulnerability and management indexes. CCA explained 61.0% of variation of management intensity, mainly by socio-cultural factors (30.32%), whereas ecological data explained 7.6% and the intersection of all factors 21.36%.

**Conclusions:**

The highest vulnerability was identified in wild species restrictedly distributed and/or highly extracted. Social pressures may increase the natural vulnerability of some species and these species are particularly those native species receiving some management form.

## Background

The subfamily Agavoideae of the plant family Asparagaceae includes species native to the Americas, naturally distributed from southern USA to Bolivia and the Caribbean Antilles [1]. The most diverse genus of the subfamily is *Agave*, which includes 204 species [2]. A total of 163 agave species occur in Mexico, 123 of them being endemic to the territory of this country, a reason why the area is considered a main centre of origin and diversification of the genus [2-6]. The *Agave* species have great ecological importance in Mexico since they are main components of arid and semiarid ecosystems predominating in most of the Mexican territory. Also, agaves are of high cultural and economic importance in Mexico since they have been used by Mesoamerican peoples from prehistoric times [7,8], and currently are valuable non-timber forest products that increasingly provide direct goods and monetary incomes to thousands of rural families. Agaves are crucial plant resources for most Mexican native cultures.

Our study focused on the *Agave* species of the Tehuacán-Cuicatlán Biosphere Reserve (ahead shortly referred to as the Tehuacán Valley), a region located in the south-eastern portion of the state of Puebla and the north-western part of the state of Oaxaca, in central Mexico. This region harbours the highest richness of *Agave* species in México [2], and is therefore particularly important to study both ecological and human cultural aspects of this plant group. We analysed the diversity of uses of all the species identified, their cultural and economic importance, and ecological information in order to examine the vulnerability of species and populations associated to use intensity of their products. In addition, we systematized the local experience of managing agaves in the region. We analysed the relation between risk and management responses to decrease vulnerability of particular species, and the viable perspectives of sustainable management to decrease risk of the species studied based on traditional ecological knowledge and technology.

A total of 34 *Agave* species has been recorded in the Tehuacán Valley, 25 species and four infra-specific taxa being native to the region, while seven of them are endemic to the area [2,9]. Our previous ethnobotanical studies carried out in the region have documented that more than 20 of all agave species of the region are used by local peoples in at least one of several use types [10]. The history of interactions between people of the Tehuacán Valley and agaves is nearly 12,000 years old [8], and such a long history allows supposing the existence of a deep traditional ecological knowledge (TEK) accumulated by the local cultures until present. The TEK of agaves by local peoples includes topics such as use properties of their parts that make them suitable for different purposes, information about distribution and abundance, particular habitats, interactions with other organisms (flower visitors, herbivores, nurse plants, parasites and pests), reproduction types, phenology, as well as management techniques that were and are continually developed and transmitted among human generations [10,11].

Uses reported for agaves in the region include live fences, food, fibre, fuel, material for construction, medicine, fodder, and alcoholic beverages such as ‘pulque’ (a sap fermented kind of wine) and ‘mescal’ (a distilled spirit) [2,10,12-17]. Some studies have documented that agave products represent a significant contribution to sustenance of rural households of the Tehuacán Valley [10,12,16,18,19]. However, the extraction of their products is frequently practiced without mechanisms directed to ensure their maintenance for future availability. The over-exploitation of some agave species has increased relatively recently apparently in direct relation with the demand of their products in the markets, which also increases the risk of their extinction [20,21]. Sustainable forms of use of these resources are currently indispensable, and because risk is determined by complex interactions of socio-cultural, institutional, biological, climatic and geographic factors and processes, the sustainable management have to take into account those factors and processes. This study analyses some of the main factors affecting risk and sustainability of *Agave* species of the Tehuacán Valley, an information that may be useful for designing both sustainable use and conservation policies.

The production of alcoholic beverages is particularly important in some villages of the region, but agave extraction involved in this activity determines high risk of maintenance of wild populations of the *Agave* species used and the biotic communities they form part. It is common that extraction of agaves from wild populations occurs without planning and with limited actions directed to recover and conserve the populations affected. These are for instance the cases of *A. potatorum* and *A. marmorata*, which are the wild species mostly used for producing mescal from whose populations’ individual plants are extracted before producing their inflorescences, thus interrupting their sexual reproduction. Another extreme of inadequate management may also be illustrated in cultivated species such as *A. angustifolia*, which in Tehuacán is managed similarly to the industrial production model of the tequila agave (*A. tequilana* var. *azul*) in other areas of Mexico [22-24]. Plantations of *A. angustifolia* progressively substitute forest areas through vegetative propagules of relatively few clones, thus determining impoverishment of vegetation composition and genetic diversity of the cultivated populations of agave.

Ethnobotanical studies have documented a high diversity of strategies of plant management in the Tehuacán Valley, including several forms of gathering, silvicultural systems that involve the management of individual plants, populations and biotic communities, as well as agricultural systems [11,12,25-28]. Silvicultural management techniques involve the management of wild populations, commonly in forest and agroforestry systems [12,27,29]. These practices are exceptionally important since they can be implemented in biodiversity conservation programs at either local or regional scales, as well as programs for wellbeing of the regional human population.

Populations of several agave species are under different management regimes in the region, the management types being influenced by ecological, socio-cultural and technological factors. Management includes human interventions in order to achieve a balance between the amount of products required to satisfy human needs and the availability of plant or animal resources. Commonly, the interaction between people and plants determines an impact that depends on the intensity of extraction of products and the technique implemented to obtain the products. The form of this interaction may represent risks to the viability of the plant resources associated to its use. For instance, practices of extraction may involve different plant parts (leaves, flowers, inflorescences or entire individuals), different amounts (for direct consumption by households or for commercialization), affecting differently the capacity of a species to tolerate the harvest of its products [30] and their recovering. Socio-cultural factors may influence fluctuations in the demand of products or their importance in satisfying a cultural requirement, which may also be according to the substitutability of a product for other. These aspects may influence the intensity of the forest products extraction and may be variable throughout time according to cultural, social and economic changes.

Some rural communities have constructed agreements and rules for regulating the access to products of some agave species. These regulations may contribute to mitigate negative impacts of forest extraction and influence the level of organization of the communities for planning the use of plant resources. Ecological aspects like distribution and abundance of the species used may also influence the magnitude of risk. An abundant and widely distributed species has lower risk than other scarcer with a more restricted distribution. Similarly, biological aspects such as life cycle length, low capacity and slow regeneration of parts extracted may determine higher risk in longer lived plants with slow growth than in others with faster growth and shorter life cycle. The type of reproductive biology is also important, since agaves with vegetative propagation are more easily recovered than those without this propagation type; those species with self-compatible breeding systems or with generalist pollinators may more surely achieve sexual reproduction than self-incompatible and/or with specialist pollinators. All these aspects influence the degree of vulnerability of the utilized species.

In this study we report ethnobiological and ecological information collected through our studies in relation to distribution, abundance, use and management strategies of the agave species in the Tehuacán Valley. We examined the hypothesis that management strategies are proportional to the degree of risk determined by ecological and social factors and that the purpose of such management strategies is to decrease vulnerability. To test this hypothesis we constructed an index of vulnerability integrating information for biological, ecological and social indicators collected for the different species of *Agave* of the region. We looked for identifying the most vulnerable species and the most relevant factors influencing such vulnerability. Accordingly, our study aimed at: 1) integrating ecological and ethnobotanical information of useful agave species in the Tehuacán Valley, in order to analyse their current state of vulnerability and its relation to management types, and 2) discussing conservation and sustainable management policies of agave species at regional scale based on local TEK.

## Materials and methods

### Study area

**Figure 1:**
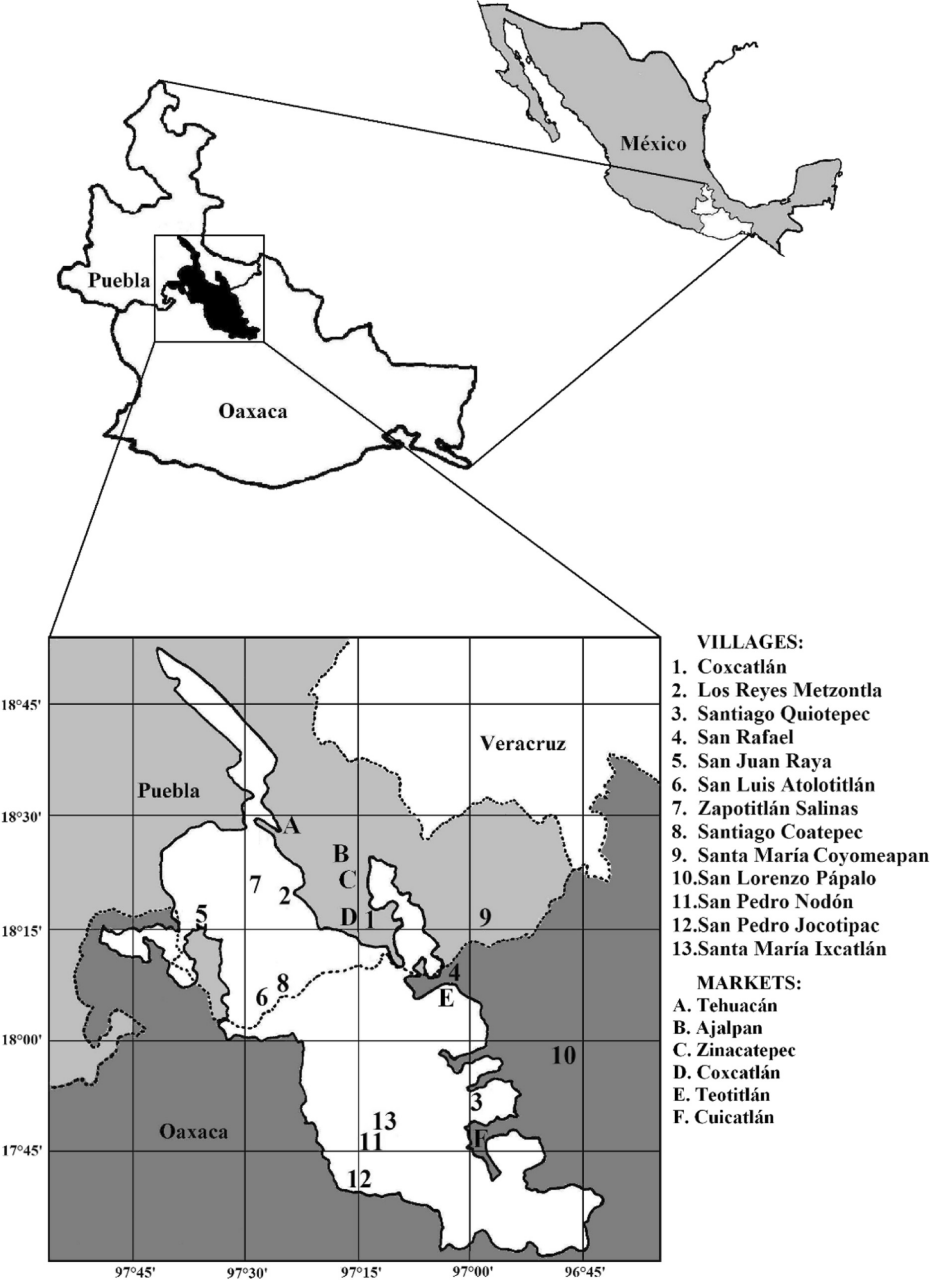
**The Tehuacán-Cuicatlán Biosphere Reserve in central Mexico.** Location of the studied communities and regional markets.

The Tehuacán Valley is the southernmost semiarid area of Mexico [31], located in the south-eastern portion of the state of Puebla and the north-west of Oaxaca. The total area is 10,000 km^2^, with the Biosphere Reserve Tehuacán-Cuicatlán covering 4,300 km^2^ (Figure 1). It is delimited by the mountains of the Sierra de Zongolica which determines the rain shade influencing the dryness of the area [32]. Climates may be warm with annual precipitation of 700 to 800 mm in the southeast, semi-warm with annual average precipitation of 400 to 500 mm in the central and western zones, and temperate with higher annual precipitation in the highlands [33,34]. This region is one of the main reservoirs of biological diversity of the arid zones of México, including more than 3,000 species of phanerogamic plants [32]. Ethnobotanical studies [10,16], have reported more than 1,600 plant species utilized by local peoples of eight indigenous ethnic groups inhabiting the region [10].

The Tehuacán Valley comprises a wide variety of environments determined by climates, soils, geomorphology and elevation, which is reflected in nearly 36 types of plant associations as described by Valiente-Banuet et al. [35,36].

### Distribution of *Agave* species

In order to analyse distribution of agave species within the territory of the Tehuacán Valley we recorded their presence in all vegetation types described by Valiente-Banuet et al. [35,36]. We have recorded data of abundance (density and biomass) in some of those areas through vegetation sampling. But since information is incomplete for all vegetation types and all agave species we did not include this information in the current analysis.

### Ethnobotanical and vulnerability studies

**Figure 2:**
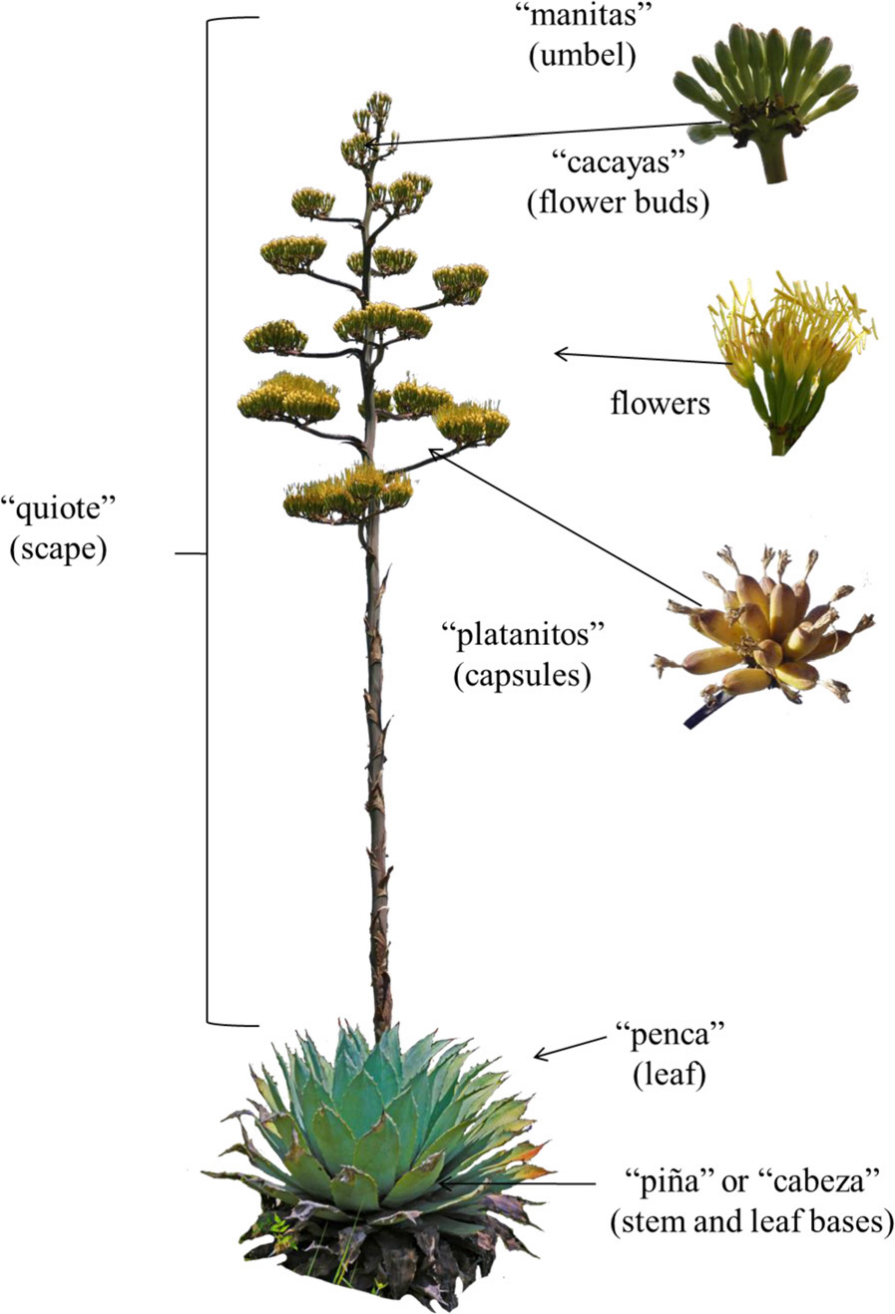
**Parts of the agave plants analysed in this study.** Common names recognized by local people in the Tehuacan Valley and the botanical names of plant parts are included.

Ehnobotanical information about use types and plant parts utilized (Figure 2), management, cultural and economic values was collected in a total of 13 rural communities and six regional markets (Figure 1). In the communities studied, a total of 176 persons provided information about agaves in a period of eight years. These were general ethnobotanical studies in relation to useful flora of the communities and the information is comparable. These studies were conducted through ethnobotanical collecting and semi-structured interviews which allowed contextualizing use of agaves in the different aspects of life by local people, in the general context of the role of plants as provider of food, medicines, live fences, fuel, fibres, among other uses. In each study, we selected at random 20 households per village but not always was recorded information about agaves.

Ethnobotanical studies were particularly directed to obtain deeper information about *Agave potatorum*, *A. marmorata* and *A. salmiana*, three species identified to be in particular high risk, as well as in relation to all agave species. The specialized studies on agaves included focal groups with nearly 80 persons in two villages (San Luis Atolotitlán and Santa María Ixcatlán), and samples of 36 informants that participate in different aspects of production of mescal. The deeper studies on agave allows a more precise relative hierarchical information about the perception of the importance of agaves by local peoples. The specific interviews were directed to deeper information about use and forms of preparation and particularly about management techniques and relative comparisons of the cultural and economic importance of the products provided by agaves. Finally, interviews in the markets were directed to identify amounts, frequency, prices, demand of products of agave species in the regional markets. Studied of commercialization of agave products in the six main markets of the region of the Tehuacán-Cuicatlán Valley in a period of two years, to 40 persons that sell mescal, flower buds and other useful products of agaves in the markets. The information is complementary and allows a general view of the importance of agaves in the regional context.

A data base was constructed in order to systematize the ethnobotanical and ecological information about distribution and biological information about the reproductive system, the length of life cycle, among the most meaningful features. The vulnerability of agave species was determined in a relative scale based on the impact of parts collected and intensity of extraction according to the criteria explained in Table 1.

**Table 1:** **Criteria used to define the values of vulnerability of *Agave *species according to socio-cultural, economic, ecological, and biological indicators**

**Variable**	**Criterion**	**Value**
**Useful part**	Use of dead plant parts	0
	Use of vegetative parts (leaves, fiber, spines)	1
	Use of sap and reproductive parts (flower buds, inflorescences	2
	Use of the entire plant	3
**Management**	Cultivated, domesticated, and introduced species (no wild populations occurring in the region)	0
	Wild native species cultivated *ex-situ* by seeds	1
	Wild native species cultivated *ex situ* through vegetative propagules	2
	Wild native species tolerated and protected in situ in modified originally natural areas	3
	Wild native species under simple gathering of vegetative parts (leaves) and vegetative sprouts.	4
	Wild native species under simple gathering of reproductive parts (flowers and inflorescences) and entire individual plants before sexual reproduction	5
**Demand in markets**	Not interchanged in markets	0
	Commercialized or bartered in markets	1
**Ecological status**	Cultivated introduced species	1
	Wild and cultivated species	2
	Only wild populations	3
**Propagation**	Seeds, caespitose and rhizomatous suckers and/or bulbils	1
	Seeds and multiannual rhizomatous suckers	2
	Seeds and low production of early rhizomatous suckers	3
	Seeds and axilar suckers	4
	Exclusively seeds	5
**Distribution in regional vegetation types**	Occurring in five or more vegetation types	1
	Occurring in four regional vegetation types	2
	Occurring in three regional vegetation types	3
	Occurring in two regional vegetation types	4
	Occurring in one single regional vegetation type	5
**Distribution in other regions of Mexico**	Cultivated broadly distributed species	0
	Occurring in more than six states of Mexico	1
	Occurring in two to five states of Mexico	2
	Endemic to the region	3

### Data analyses

In order to identify the different conditions of vulnerability based on variables related with social and ecological risk, a principal component analysis (PCA) was performed. The vulnerability index was determined as the score of the first principal component, which is an integration of information of the most meaningful variables analysed. Similarly, different indicators of plant management were analysed through PCA and the score of the first principal component was used as index of management intensity. The relation between vulnerability and management intensity was analysed through a regression analysis. Amounts of variation of management data explained by ecological, cultural and economic information, as well as their vulnerability level were analysed through canonical correspondence analyses (CCA) CCA were performed to measure the amount of variation of management data explained from ecological and sociocultural information. The analyses were conducted using the R software following Blancas et al. [28]. We used three matrices partitioning the variation: Matrix Y containing the response variables (management intensity data matrix), matrix X with the set of explanatory ecological variables; and matrix W with the set of explanatory sociocultural variables. The main purpose of this analysis is to cope with the confounding effects that may occur if a CCA of Y is made using W or X as the only explanatory matrix. That is, some variables of W may influence variables of X and vice versa. Through this method we conducted several CCA combining sets of explanatory variables: 1) Correspondence Analysis (CA) only for matrix Y, 2) CCA for matrix Y vs. matrix W, 3) CCA for matrix Y vs. matrix X, 4) CCA for matrix Y vs. matrices W+X. The total constrained eigenvalue of each analysis was tallied to identify how much of the management intensity matrix is explained by ecologic and sociocultural data. This method allowed dividing CCA variation into four parts: a) Ecological data, which is the fraction of management intensity variation that can be explained by ecological data independently of sociocultural data, b) Sociocultural + ecological data, c) Sociocultural data which is the fraction of management intensity variation that can be explained by sociocultural data independently of ecologic data, and, d) Undetermined data or fraction of management intensity variation explained neither by ecological nor by sociocultural data. For each of these analyses, the sum of all canonical eigenvalues divided by the sum of all canonical eigenvalues, allowed calculating the corresponding fraction of variation explained by the analysis. Significance of the models for each CCA was estimated by permutation tests for: a) the whole model, b) management intensity explained by ecological variables and 3) management intensity explained by sociocultural variables.

## Results

### Uses

**Figure 3:**
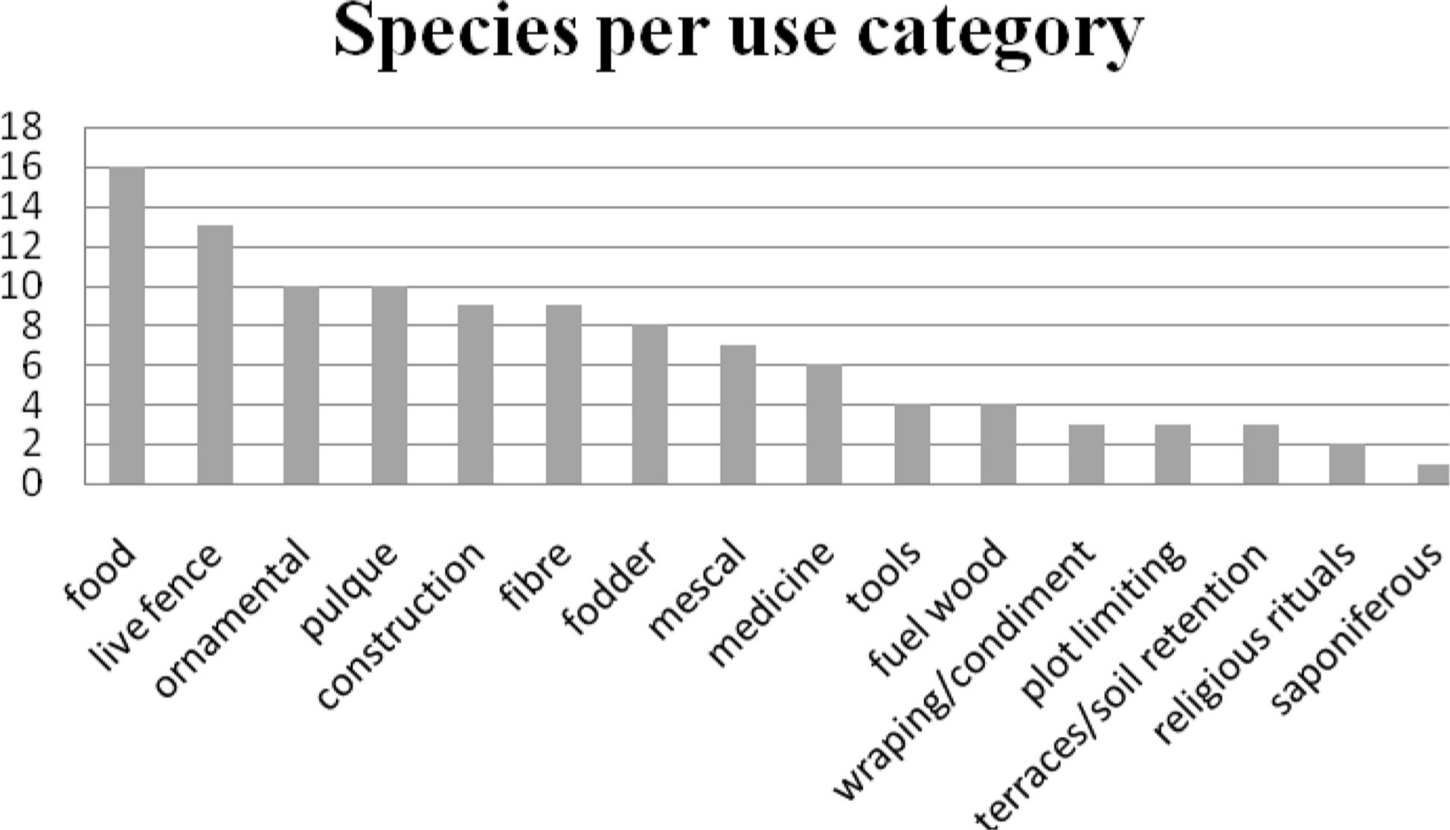
**Number of *Agave *** **species (ordinate axis) of the Tehuacán Valley identified in this study with different use types.**

*Agave* species are strongly rooted in cultures of local peoples of the Tehuacán Valley. From a total of 34 species recorded 28 have at least one use. Table 2 summarizes information on the variables used for analysing the ecological, social and cultural aspects for each species studied. *Agave marmorata* is the species with the highest number of uses (14), followed by *A. potatorum* with 12. Nearly 25% of the species analysed have one single use (Figure 3). The main uses of agave documented in this study are:

**Table 2:** **Information about ecological, socio-cultural and management aspects of the useful *Agave *species recorded in the Tehuacán Valley**

**Species**	**Ecological status**	**Vegetation types occurrence**	**Distribution**	**Life cycle**	**Reproduction forms**	**Management types**	**Sites proximity**	**Collective regulations**	**Artificial selection**	**Use types**	**Used parts**	**Harvest type**	**Commercial value**	**Medicinal use**
*A. americana* var. *americana*	1	0	0	1	2	2	0	1	2	2	2	2	1	0
*A. a.* var. *marginata*	1	0	0	1	2	2	1	1	2	1	0	2	1	0
*A. a.* var. *oaxacensis*	1	0	1	1	2	2	1	1	2	2	3	2	1	0
*A. angustiarum*	3	3	1	1	1	5	0	1	1	3	2	1	0	0
*A. angustifolia*	1	0	1	1	2	3	1	1	2	2	1	1	1	0
*A. angustifolia var. angustifolia*	1	4	0	1	2	2	1	1	2	1	2	2	1	0
*A. applanata*	2	3	1	1	2	4	0	1	1	1	1	1	0	0
*A. atrovirens*	3	2	2	1	1	3	2	1	2	1	2	2	0	0
*A. atrovirens* var. *atrovirens*	3	5	2	1	1	2	1	1	2	2	2	2	1	0
*A. atrovirens* var. *mirabilis*	2	5	2	1	1	2	1	1	2	2	2	2	0	0
*A. chiapensis*	3	0	2	2	2	4	0	1	1	1	2	1	0	0
*A. convallis*	3	5	2	1	2	4	0	1	1	1	1	1	0	0
*A. ghiesbreghtii*	3	5	2	2	2	3	0	1	1	2	1	1	0	0
*A. karwinskii*	2	4	2	1	2	2	2	1	1	2	1	2	1	1
*A. kerchovei*	3	4	2	1	2	4	2	1	2	2	2	1	1	0
*A. macroacantha*	2	4	2	1	2	5	2	1	2	3	2	2	1	0
*A. mapisaga*	1	4	0	1	2	2	1	1	2	2	2	2	1	0
*A. marmorata*	3	4	2	1	2	4	2	1	2	4	3	2	1	3
*A. nussaviorum* subsp. *nussaviorum*	3	5	3	1	1	5	0	1	1	2	3	1	0	3
*A. peacockii*	3	5	2	1	2	4	0	1	2	3	2	2	0	0
*A. potatorum*	3	3	2	1	1	1	2	2	2	4	3	2	1	3
*A. salmiana* subsp. *salmiana*	2	2	2	1	2	2	1	1	2	2	2	2	1	0
*A. salmiana* subsp. *tehuacanensis*	2	3	2	1	2	3	2	1	2	3	3	2	0	0
*A. scaposa*	3	1	2	1	1	3	0	2	2	3	0	2	0	2
*A. seemanniana*	1	0	2	1	1	5	2	1	2	3	3	2	1	1
*A. stricta*	3	2	2	2	2	4	0	1	2	2	2	1	1	0
*A. titanota*	3	0	2	1	2	5	0	1	2	2	3	2	0	0
*A. triangularis*	3	4	2	2	2	5	0	1	1	3	2	1	0	0

i. Food, particularly appreciated are flower buds (‘cacayas’) and escapes (‘quiotes’) which are prepared boiled and cooked in various stews. Also important are the stems of mature plants which are cooked in underground ovens producing a sweet fibrous meal (the product is called ‘mezcalli’). A total of 16 species were mentioned to provide edible parts (Table 1).

ii. Live fences which are mainly destined to limiting plots, protecting them to the access of livestock and to conform barriers or lines to retain soil and water; live fences with agaves are particularly important in agroforestry systems out of the villages, as well as in homegardens. A total of 13 species were mentioned to be commonly used for this purpose (Table 1).

iii. Alcoholic beverages, which are prepared as ‘wine’ or ‘beer’ by fermenting the sap, this beverage is called ‘pulque’, and it is mainly prepared with *A. americana* var. *americana*, *A. salmiana* subsp. *tehuacanensis*, *A. atrovirens*, *A. marmorata* and a total of 13 species. The distilled beverage is called mescal. It is distilled after fermenting the cooked stems, and the most important species used in the region are *Agave potatorum, A. americana* var. *oaxacensis*, *A. macroacantha*, and *A. marmorata*, and other species, seven in total.

iv. Fibre, which is used as raw matter to manufacture cords or ‘yute’ and ‘ixtle’; these materials in the pre-Columbian times were as important as cotton for netting and clothing. A total of 11 species are currently used for obtaining fibre.

v. Ornamental. In total, 11 agave species are used as ornamental plants, the most appreciated are *A. triangularis*, *A. macroacantha*, *A. stricta*, *A. americana*, *A. marmorta*, and *A. potatorum.*

vi. Biofuel, the remains of dead adult agaves (called in Náhuatl ‘mezote’) are much appreciated as fuel. Particularly important are those large size species.

vii. Nine agave species provide material for construction, particularly the escapes of several species which are valued as wooden material for supporting roofs, walls and fences in traditional constructions. In some villages the escapes are used as pipes conducting water, whereas in other villages the leaves are also used for thatching houses and fibre is used to make cords for tiding structures of the houses. Particularly important for construction are the escapes of *A. scaposa* and *A. salmiana* var. *tehuacanensis*. For corral fences and platforms for storing stubble are preferred the smaller escapes of *A. potatorum* and *A. kerchovei*.

viii. Tools, large escapes are used for manufacturing ladders, whereas the thinner and longer escapes are used as pole vault or ‘chicoles’ used for collecting fruits from trees and columnar cacti.

ix. Fodder, the young plants and escapes in early stages of formation are consumed by livestock, mainly cattle and goats. Eight species are fodder, *A. angustiarum*; *A. angustifolia* var. *angustifolia*, *A. atrovirens* var. *mirabilis*, *A. kerchovei*, *A. macroacantha*, *A. marmorata*, *A. potatorum* and *A. scaposa*.

x. Medicine, six agave species are medicinal. Their roosted leaves are anti-inflammatory and analgesic used to relieve luxation pain; leaves infusions are used for bronchitis and as anti-inflammatory of internal organs, as well as anticoagulant. The cooked stem called “mezcalli” is used for bronchus affections. The alcoholic beverage called mescal is used as other spirituous beverages, for relieving stomach ache, tonic for whetting appetite, and drinking it with honey and lemon is used for relieving cold; it is also used rubbing feet and the back together with branches of the tree ‘pirul’ (*Schinus molle*) for relieving fever and ‘heating cold feet’. Mescal is also used to prepare tinctures with medicinal plants and poultices for treating rheumatic and traumatic pains.

xi. The leaf cuticle, called ‘mixiote’ is used to envelope food, mainly meat that is cooked underground; it is a material of high demand and price in markets. Two species were mentioned to be used for extracting mixiote.

xii. Ritual and religious, mainly for preparing altars in religious celebrations (two species).

xiii. Soap, *A. triangularis* was referred to be used as soap for washing clothes because of its high content of saponins.

### Useful parts

In the case of *Agave marmorata* we found the use of six plant parts, *A. potatorum, A. seemaniana* and *A. kerchovei* provide five useful parts. In total, 17 species provide two to four useful parts and eight species provide one single useful plant part. Figure 2 shows the agave plant parts considered in the analysis, indicating their regional names and their equivalent botanical names. The entire living plants of 16 species are transplanted *in situ* or *ex situ* as living fences and borders or terraces, *A. marmorata* and *A. americana* var. *americana* are the most common. Flower buds and inflorescences of nearly half of the species analysed are used.

### Economic value

In total, 11 species were reported to have commercial value and 17 were not (Table 2). This information could be underestimated since some agave products are occasionally interchanged or bartered at local level in the villages and were not easily recorded. For instance, mescal producers interchange mescal for maize and other products in the local stores, or even for other products used for producing mescal (agave plants, fuel wood, or labour hand). The commercial value is an indicator of the amount of demand of an agave species or its product, but the risk is not necessarily a factor determining risk, it depends of other aspects as well. For instance, a household that collect flower buds for commercialization in the regional markets does not determine an impact similar to that determined by a mescal producer household, which may extract 200 individual plants for one single production event. Even lower impact can be identified in practices for collecting dry escapes for construction and manufacturing tools. In these cases the agaves have died and released their seeds and, therefore, impact being null. However, escapes of *A. marmorata* are massively cut for ornamental purposes in the village of Zapotitlán during the blooming period, and those of *A. salmiana* ssp. *tehuacanensis* are harvested in San Luis Atolotitlán, Puebla, inhibiting the development of flowers, fruits, and seeds, decreasing the possible contribution of sexual reproduction.

### Management

The following forms of interaction between local peoples and agave were identified:

(1) Out of the 28 species of *Agave* reported with some use in the Tehuacán Valley, seven are extracted exclusively from wild populations (simple gathering) whereas the remaining species receive at least one management type.

(2) The most common management type is extraction of entire individual plants while transplanting seedlings or young plants to anthropogenic areas (*ex situ* transplanting). This are the cases of *A. atrovirens* var. *atrovirens*, *A. karwinskii*, *A. macroacantha* and *A. titanota*.

(3) The practice referred to above is followed in frequency by the extraction of plants exclusively from wild populations but at the same time people may practice *in situ* propagation of propagules, as in the cases of *A. angustiarum, A. kerchovei*, *A. peacockii*, *A. stricta* and *A. scaposa*.

(4) Then, it can be mentioned the importance of the vegetative propagation of domesticated, introduced, and widely distributed species such as *A. americana* var. *americana*, *A. americana* var. *marginata*, *A. angustifolia* var. *angustifolia* and *A. mapisaga*.

(5) There is in addition the incipient cultivation of *Agave potatorum* in green houses, particularly in San Luis Atolotitlán and Caltepec, Puebla, where local people have had the initiative to produce plants for recovering wild populations. Plants produced are therefore reintroduced to the original wild areas, but they started to test their success in small plots of agroforestry systems and in abandoned agricultural areas.

(6) Finally, we recorded the intensive cultivation of plantations of *A. angustifolia* and more recently also *A. tequilana* var. *azul* which are intensively produced as monocultures in plots using agrochemicals.

(7) However we identified another management form. Extraction of agave sap for preparing ‘pulque’ is one of the most ancient uses of agaves. Plants used for this purpose are propagated through vegetative propagules, mainly to areas close to the houses in the village, as well as to areas surrounding the agricultural plots and homegardens and even along roadsides. It is an example of small scale cultivation. These are the cases of *A. americana* var. *americana* and *A. atrovirens* var. *atrovirens*.

(8) Other species like *A. stricta*, *A. macroacatha* and *A. potatorum* are cultivated in nurseries of local Units of Environmental Management (UMAs for their abbreviation in Spanish), where local people propagate seeds collected in wild populations and sell young plants as ornamental. This is part of a strategy directed to decrease the illegal extraction of plants from the biotic communities by international illegal trade industries.

### Vulnerability index, species in risk and management intensity

**Figure 4:**
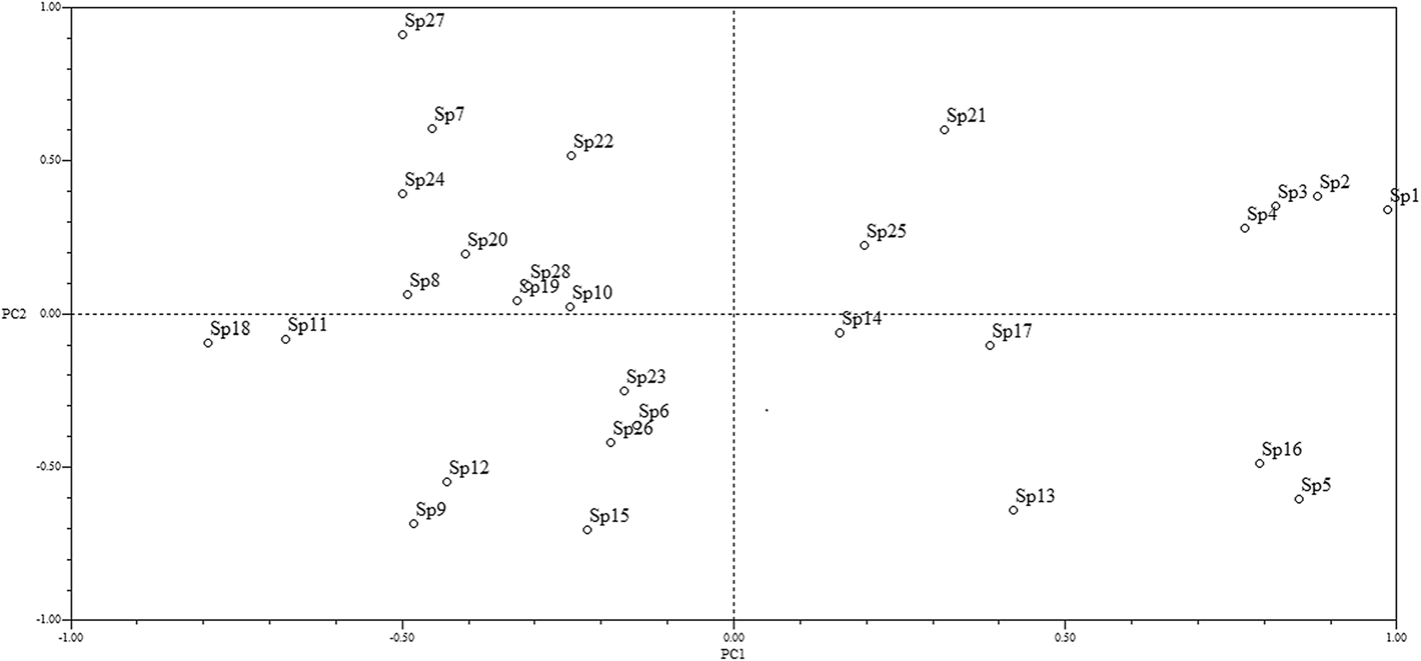
**Spatial arrangement of *Agave *species of the Tehuacán Valley according to the Principal Component Analysis performed with socio-ecological variables.** Sp1 = *Agave americana* var. *americana*, Sp2 = *A. americana* var. *marginata*, Sp3 = *A. americana* var. *oaxacensis*, Sp4 = *A. angustiarum*, Sp5 = *A. angustifolia*, Sp6 = *A. angustifolia* var. *angustifolia*, Sp7 = *A. applanata*, Sp8 = *A. atrovirens*, Sp9 = *A. atrovirens* var. *atrovirens*, Sp10 = *A. atrovirens* var. *mirabilis*, Sp11 = *A. chiapensis*, Sp12 = *A. convallis*, Sp13 = *A. ghiesbreghtii*, Sp14 = *A. karwinskii*, Sp15 = *A. kerchovei*, Sp16 = *A. macroacantha*, Sp17 = *A. mapisaga*, Sp18 = *A. marmorata*, Sp19 = *A. nussaviorum* subsp. *nussaviorum*, Sp20 = *A. peacockii*, Sp21 = *A. potatorum*, Sp22 = *A. salmiana* subsp. *salmiana*, Sp23 = *A. salmiana* subsp. *tehuacanensis*, Sp24 = *A. scaposa*, Sp25 = A*. seemanniana*, Sp26 = *A. stricta*, Sp27 = *A. titanota*, Sp28 = *A. triangularis*.

The principal components analysis classifies the agave species recognizing two main groups, one of them is composed by the cultivated agaves, domesticated or in process of domestication or recently introduced to cultivation because of their commercial value. The other group is composed by wild agaves with low or null commercial value (Figure 4). This analysis identifies the important weight of the commercial value of products in their classification (Table 3).

**Table 3:** Vulnerability and management intensity indexes estimated for the different agave species utilized in the Tehuacán Valley

**Species**	**Vulnerability**	**Management intensity**
*A. nussaviorum* subsp*. nussaviorum*	1.83122	1.708526199
*A. peacockii*	1.1645	0.594045546
*A. convallis*	1.04956	1.126756957
*A. potatorum*	0.91661	−0.502843702
*A. triangularis*	0.89015	1.801632007
*A. titanota*	0.82558	0.380721625
*A. atrovirens*	0.73993	−0.136474466
*A. kerchovei*	0.5566	0.12302843
*A. angustiarum*	0.45358	0.923424365
*A. scaposa*	0.43271	0.098578612
*A. marmorata*	0.41184	0.12302843
*A. stricta*	0.40192	0.925191476
*A. ghiesbreghtii*	0.3376	1.512068797
*A atrovirens* var. *mirabilis*	0.30958	−0.229081914
*A. chiapensis*	0.25838	1.298744876
*A. atrovirens var. atrovirens*	0.24885	0.113410498
*A. salmiana subsp. tehuacanensis*	0.20267	−0.483614095
*A. macroacantha*	0.01962	−0.034892874
*A. seemanniana*	0.00464	−0.611475086
*A. applanata*	−0.06403	0.312134623
*A. salmiana subsp. salmiana*	−0.58286	−0.552045154
*A. karwinskii*	−0.67297	−0.055894786
*A americana var. oaxacensis*	−1.3218	−1.366667488
*A. mapisaga*	−1.4866	−1.361323377
*A. angustifolia*	−1.48899	−1.182096381
*A. angustifolia var angustifolia*	−1.61646	−1.361323377
*A. americana var. americana*	−1.79597	−1.483920345
*A. americana var. marginata*	−2.02585	−1.6796394

**Figure 5:**
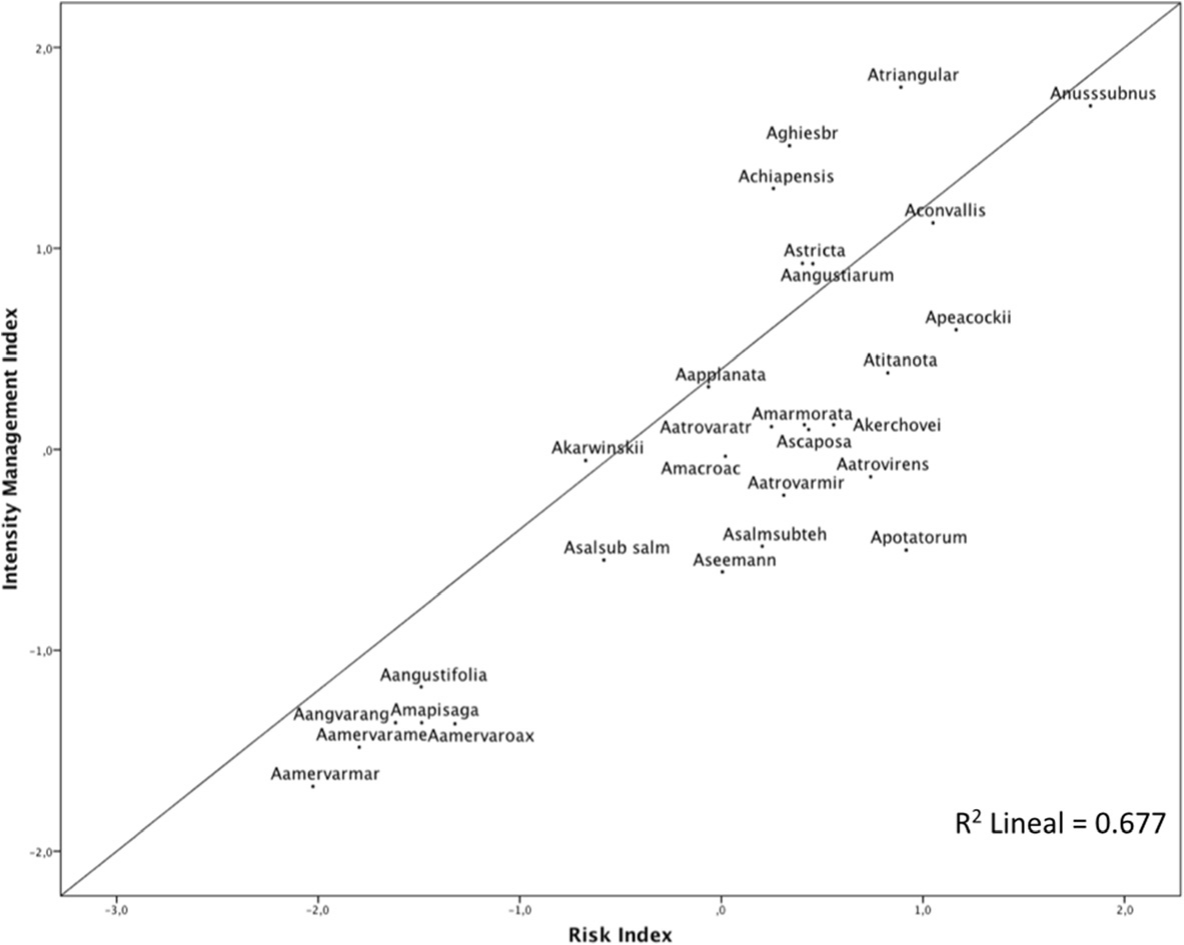
**Regression analysis of the management intensity index as a function of the vulnerability index calculated as the scores of the first principal componente of the PCA of ecological, socio-cultural and management factors studied (R**^**2**^ **= 0.677, P < 0.000).**

**Figure 6:**
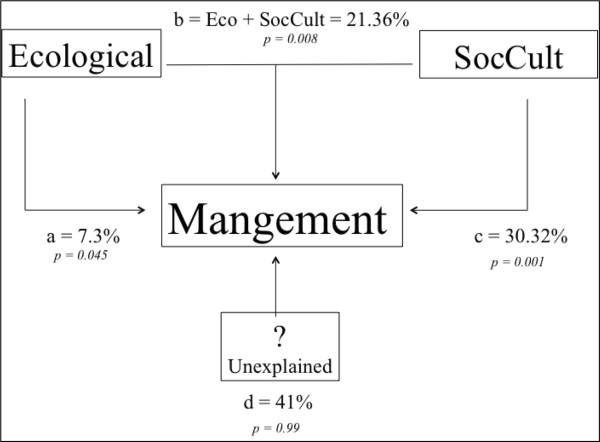
Partitioned CCA scheme showing the relative influence of ecological, sociocultural (SocCultEco) factors and their interaction on management strategies of agave plant species of the Tehuacán Valley.

The regression analysis in Figure 5 indicates the highly significant linear relation between risk and management intensity indexes (R^2^ = 0.677, P < 0.001). Partitioning CCA explains 61.0% of the management variation as shown in Figure 6. This variation can be explained mainly by sociocultural factors (30.32%) while ecological data explain 7.6%. Intersection of ecological and sociocultural factors explains 21.36% and is statistically significant. Unexplained variation was 39.0%. Four variables of the intersection of ecological and sociocultural indicators were particularly important: ecological status, life cycle, type of harvest and interchange or not of the agave products (Table 4).

**Table 4:** Permutation test for CCA variables

**Management, ecological and sociocultural factors variable**	**Df**	**Chisq**	**F**	**Pr(>F)**	**Significance**
Ecological status	1	0.0204	5.1775	0.018	*
Vegetation types where species occurs	1	0.0047	1.1888	0.301	
Distribution	1	0.0101	2.5752	0.08	
Life cycle	1	0.0144	3.6426	0.047	*
Use types number	1	0.0032	0.8036	0.449	
Utilized parts	1	0.0026	0.6683	0.508	
Type of harvest	1	0.0218	5.5248	0.009	**
Commercial value	1	0.0221	5.6139	0.01	**
Medicinal uses	1	0.0028	0.7027	0.478	
Residual	18	0.0709			
**Management and sociocultural factors**					
Use types number	1	0.005	1.3204	0.241	
Utilized parts	1	0.005	1.3281	0.261	
Type of harvest	1	0.0504	13.2815	0.001	**
Commercial value	1	0.026	6.8539	0.007	**
Medicinal uses	1	0.0029	0.7589	0.471	
Residual	22	0.0836			
**Management and ecological factor**					
Ecological status	1	0.0204	3.8029	0.032	*
Vegetation types where species occurs	1	0.0047	0.8732	0.369	
Distribution	1	0.0101	1.8915	0.159	
Life cycle	1	0.0144	2.6756	0.08	
Residual	23	0.1234			

* = P<0.05 and ** = P<0.01.

Figure 5 shows that the most vulnerable agave species are *Agave nussaviorum* subsp. *nussaviorum*, *A. peacockii, A. convalis, Agave potatorum* and *A. triangularis*, most of them with restricted distribution or intensively extracted. Those with intermediate vulnerability are *Agave marmorata*, *A. titanota*, *A. atrovirens* var. *atrovirens*, *A. scaposa*, *A. salmiana* subs. *tehuacanensis*, *A. salmiana* subsp. *salmiana*. The least vulnerable species are *Agave mapisaga*, *A.angustifolia* var. *angustifolia, A. americana* var. *americana* and *Americana* var. *marginata* which are domesticated, introduced and widely distributed.

## Discussion and conclusions

### Use and management

In the Tehuacán Valley, the species of agave are among the more used and appreciated plant resources and also those more intensively managed by people of the region. Most of them are resources located in communal land and therefore of free access to local people. Different parts of the plants are used which are available mainly during the dry season when most of the wild plant resources are not available. The high cultural and economic importance of their products and their intensive extraction determine that some species, particularly those that require using the entire plant, have also high risk of disappearing. It is possible to identify a delay in constructing agreements and institutions regulating the access to agave products; this is probably a fact related to the relatively recent and rapid pressure developed on agave resources. According to local people, local agave populations that are now extinct were abundant until recently (nearly 40 years ago). This pattern is particularly clear in those species with high commercial value like *Agave potatorum*, a species appreciated for preparing mescal with increasing demand in the regional and national market. The pattern contrasts also with that of other species mainly used to satisfy needs of direct consumption by households, and even more with those species whose use is being lost, as it is the case of the ‘pulque’ agaves like *Agave salmiana*, since pulque has been progressively substituted by more prestigious beverages like beer [28]. A similar situation can be observed in some species that in the past were used for extracting their fibres like *Agave angustiarum, Agave peacockii* and *Agave angustifolia*. Their fibre has been substituted by plastic cords and these species are now mainly used for producing mescal, which is a more profitable activity.

Regulations of access and extraction of common wild populations were recorded for some species, particularly those that have several uses. In those cases people have developed strategies that include regulated extraction in different seasons of the year. This is for instance the case of the extraction of the escapes of *Agave salmiana* subsp*. tehuacanensis* used for construction of houses which in San Luis Atolotitlán, Puebla are extracted in two or three specific days, according to the community extraction agreements.

From the analysis of vulnerability, it is clear that while more forms of management are practiced the vulnerability decreases. Management of agaves may occur in the wild (*in situ* including tolerance), as well as in anthropogenic environments (*ex situ*, including transplanting of young plants and seeds sowing). Particularly important are the agroforestry systems in the field, located out of the villages [29,37,38], where people practice a mixture of *in situ* and *ex situ* management techniques. But also important are homegardens, where people cultivate *ex situ* several species of *Agave* either wild or domesticated. The agroforestry systems in the field and homegardens are viable options for propagating agaves, not only with lower environmental impact than the intensive agave plantation for commercial tequila and mescal, but also for planning conservation of rare species and genotypes.

### Vulnerability index

The cluster (Figure 4) and principal component (Figure 5) analyses performed consistently show the importance of the commercial value for explaining the vulnerability of the agave species studied, the higher the economic value the higher the risk. But this is also a function of the distribution (the higher the distribution the lower the risk), abundance (the scarcer the resources the higher the risk) and the status of management as discussed ahead. Those exclusively wild species have higher risk than those wild regulated or *in situ* managed species; have even more risk than those that are wild and cultivated and even more than those that are exclusively cultivated. Figure 5 shows the place of each of the 28 species of *Agave* studied in a Euclidean space which in turn reflects a management gradient.

The vulnerability index (Figure 6) indicates that the most vulnerable species are five native species with relatively intermediate distribution, except *Agave nussaviorum* subsp. *nussaviorum*, which has restricted distribution and is the most vulnerable species of all the species analysed. The five most vulnerable species are extracted exclusively from the wild, although for *Agave potatorum* some practices of incipient management have started to be carried out such as the protection of populations through communitarian regulations or cultivation for being reintroduced in their natural habitats. But these practices are recent, carried out by some persons without interchanging experiences to each other. Among the species with intermediate vulnerability we identified both wild and cultivated agaves of relatively broad distribution. Almost all of these species, with the only exception of *A. scaposa*, provide reproductive parts or the entire plant as useful product, determining high risk [37] which in part is counterbalanced through the strategies of asexual reproduction in addition to propagation through seeds. *A. scaposa* reproduces exclusively by seeds but its useful product (dry escapes) are used after reproduction. Among the least vulnerable species we identified those domesticated, introduced and broadly distributed.

The vulnerability index of Figure 6 provides useful information for guiding actions and regulations in order to protect endangered agave species, as well as factors that should be taken into account for designing and implementing policies. For instance, it has been discussed some aspects to be considered when using *Agave* species with different degrees of vulnerability. From our current analysis it is clear the importance of documenting socio-cultural factors together with ecological information, particularly their distribution, abundance and type of reproduction. This information provides the basic data for designing strategies for their sustainable use.

It is also crucial that any strategy implemented could be periodically monitored in order to consider uncertainty and surprises associated to the complex socio-natural systems involved in agave management, but also in order to progressively improving the management based on previous experiences. Most agave species are exclusively wild and *in situ* management of populations is particularly important; but several regional experiences have demonstrated that a combination of germinating seeds in controlled conditions and nursing of young plants may be effective to increase the amount of plants in relation to the amount of available seeds. However, the local experience also reveals that reintroduction of plants into natural habitats requires ecological information about the interaction with other natural nurse plants that provide micro-environments that are crucial for successful establishing of young plants. For some species, plantations in degraded soils are not only possible but also one way to recover ecosystem functions while providing useful products and monetary incomes to local people.

Below, we provide some general recommendations in order to promote more sustainable forms of management of agaves in the Tehuacán Valley.

#### Extraction planning

Monitoring species and their populations available within a territory provides the basis for planning actions. This panorama makes possible identifying areas for carrying out the use of products during different production seasons and years. Some experiences in Chilapa, Guerrero, Mexico have documented the organization of communitarian committees with the charge of planning and monitoring extraction of useful products as well as actions for recovering the affected populations [39]. In some cases the proportion and relation of wild and cultivated areas should be considered in the monitoring activities since for some species wild and cultivated populations are sympatric.

#### Wild populations

Counting of reproductive individual plants and identifying and labelling the number of escapes that have to be respected when extracting wild products is necessary in order to ensure a minimum of seeds that are required for both natural and artificial propagation of agave plants. A method similar to that mentioned here has been practiced by the communities of Guerrero, Mexico in areas producing mescal (the Asociación de Magueyeros y Mezcaleros del Chilapan) [39] and their experience may be replicated in other areas for managing mescal agaves. Additional suggestions for more effective recovering of populations are:

• Collecting seeds from several sites in order to ensure diversity of sources of genetic material. This practice may favour the availability of plant material from several areas as well as options for adapting the propagated material to establish in different environments. Production of plants in nurseries may optimize the production of plants from relatively few seeds, but this practice should be complemented with dispersion of seeds under the canopy of natural nurse plants. Where survival of young plants may be more successful than those transplanted from nurseries.

• Transplanting of young plants of agave under the canopies of specific nurse plants recognized by previous ecological research available in the literature. In addition it is recommendable to conduct experiments to test the successful establishing of seedlings and young plants under artificial shade.

• Recovering populations of agaves in areas identified as areas of extinction of local populations, as well as in areas where populations are being impacted by human activities, and finally as plantations in areas that were cleared for agriculture in the past. These areas may be benefited from soil recovering associated to presence of agaves. Mescal production could be centred in these areas rather than form populations from natural forests.

#### Nurseries

Rustic local materials can be used for preparing nurseries. In Sola de Vega, Oaxaca, the mescal producers of the Sociedad de Producción Rural el Solteco produce *A. potatorum*, *A. angustifolia* and other species in rustic wooden beds in areas thatched with shrubs and palm leaves.

#### Cultivated species

Young plants may be produced from seeds collected in the region or, in some species, from vegetative propagules. Interchange of seeds and vegetative propagules from different zones is recommendable in order to increase options for different purposes and environments where the plant material will be propagated. It is highly recommendable experimenting transplantation of young plants in agroforestry systems either in the field out of the villages [29,37,38] or in homegardens. These systems are recognized to harbour high proportions of natural vegetation and, therefore, this practice may reinforce the role of biodiversity conservation of these systems. Plantations should be directed to recover plant cover of deforested and eroded areas. However, this task is still particularly difficult and more techniques are still needed to be developed.

#### Protection of pollinators

Bats are the most important pollinators of most agave species of the Tehuacán Valley. Unfortunately, in the past some governmental agencies promoted campaigns for eradicating them with the erroneous idea that bats promoted illnesses. It is therefore necessary to promote campaigns for protecting bats which are crucial for pollination of agaves and numerous other groups of plants. Reproductive success of species that entirely depend on sexual reproduction for their viability (*A. convallis*, *A. nuusaviorum*, *A. peacockii*, *A. potatorum*, *A. scaposa* and *A. seemanniana*) will in turn depend on the success of protection of bats and other pollinators.

Survival and permanence of agave populations depend mainly on actions of those that use them. However, authorities of the Biosphere Reserve may provide support in numerous campaigns of information and favouring fair commerce of products. Regional policies for sustainable use of local resources are possible and necessary and local authorities have a clear responsibility to achieve this task. The academic sector that carry out research in the region should be more active in directing their studies to understand key aspects that are necessary for designing sustainable forms of management of non-timber forest products without forgetting the thousands of years of experience of local people. Scientific research may be extraordinarily valuable tool when complementing initiatives by local peoples.

## Competing interests

The authors declare that they have no competing interests.

## Authors’ contributions

AD-L main author, involved in the study design, conducted interviews, field work, literature review and general data collection and systematization, wrote the first draft and concluded the final version of this manuscript. IT and JB contributed to designing and following progress of the research and field work and data analyses. AC main coordinator-supervisor of the research project; contributed with original data and the designing of all the researches providing the information for the current analysis; participated in fieldwork, systematization and analysis of data and reviewed several drafts of the manuscript. All authors read and approved the final manuscript.

## Authors’ information

AD, IT, JB postgraduate students at the Centro de Investigaciones en Ecosistemas (CIEco), UNAM. AC full time researcher at CIEco, UNAM.
